# Twins Support the Absence of Parity-Dependent Fertility Control in Pretransition Populations

**DOI:** 10.1007/s13524-020-00898-0

**Published:** 2020-07-17

**Authors:** Gregory Clark, Neil Cummins, Matthew Curtis

**Affiliations:** 1 Department of Economics, University of California, Davis, Davis, CA USA; 2 Department of Economic History, London School of Economics, and Center for Economic Policy Research, London, UK

**Keywords:** Fertility, Family planning, Natural fertility, Economic history, Economic growth

## Abstract

A conclusion of the European Fertility Project in 1986 was that pretransition populations mostly displayed *natural fertility*, where parity-dependent birth control was absent. This conclusion has recently been challenged for England by new empirical results and has also been widely rejected by theorists of long-run economic growth, where pre-industrial fertility control is integral to most models. In this study, we use the accident of twin births to show that for three Western European–derived pre-industrial populations—namely, England (1730–1879), France (1670–1788), and Québec (1621–1835)—we find no evidence for parity-dependent control of marital fertility. If a twin was born in any of these populations, family size increased by 1 compared with families with a singleton birth at the same parity and mother age, with no reduction of subsequent fertility. Numbers of children surviving to age 14 also increased. Twin births also show no differential effect on fertility when they occurred at high parities; this finding is in contrast to populations where fertility is known to have been controlled by at least some families, such as in England, 1900–1949, where a twin birth increased average births per family by significantly less than 1.

**Electronic supplementary material:**

The online version of this article (10.1007/s13524-020-00898-0) contains supplementary material, which is available to authorized users.

## Introduction

We propose a new test, based on parents’ response to the accident of a twin birth, of whether pretransitional populations were practicing parity-specific fertility control. We apply this quasi-experimental test to four micro-demographic data sets: the well-known family reconstitution studies of Enquête Louis Henry and CAMPOP, a large genealogical database from Québec, and a novel genealogical database from England. (Find more on all in the upcoming Data section.) In sum, we analyze the effects of 16,580 twins on 709,262 births and confirm that for English marriages pre-1880, French marriages pre-1789, and Québec marriages pre-1830, there was no sign of significant parity-specific fertility control.

Why is such a test needed? After all, historical demographers, by the 1980s, concluded that parity-specific fertility control was absent from most pretransition populations (Coale 1971; Coale and Trussell 1974, 1978; Coale and Watkins 1986; Henry 1953, 1961; Knodel 1974, 1978, 1983; Knodel and Walle 1979).^1^

This test is needed for two reasons. First, in contradiction to this prior research, a literature has emerged claiming to establish that even in the pretransition era, there is strong empirical evidence of parity-dependent birth control. These researchers have argued that with populations that passed the traditional tests of natural fertility—for example, England pre-1850—substantial parity-dependent control does exist (Cinnirella et al. 2017, 2019). Anderton and Bean (1985), David and Sanderson (1988), Van Bavel and Kok (2004), and Kolk (2011) similarly claimed to find parity-dependent control or its like in pretransition populations in the United States, France, Sweden, and the Netherlands. These claims are possible because the methods used to establish an absence of parity-specific control in the natural fertility literature depend on untested assumptions that the very population the new literature argues consist of controllers actually was operating with natural fertility.

Another line of research has argued that pretransition populations controlled birth spacing in response to annual variations in living standards or to numbers of dependent children (Amialchuk and Dimitrova 2012; Cinnirella et al. 2017, 2019; Dribe and Scalone 2010; Kolk 2011; Van Bavel 2004; Van Bavel and Kok 2004). Such control in response to material conditions is not in itself evidence of parity-specific control. However, if there was control in response to annual fluctuations in living conditions, then families at higher net parities—who faced resource constraints equivalent to those of bad harvests—would have had both the means and the inclination to increase spacing.

Second, a significant literature in economics assumes that all pre-industrial populations exercised parity-dependent fertility control. In theorizing about the demographic transition, economic models almost universally assume that pretransition fertility was controlled fertility. Pretransition families had higher family target sizes as a result of such factors as lower child survival rates, costs of child-rearing, child earnings, and the education premium in earnings. Still, parents had target family sizes and exercised parity-dependent control (see, e.g., Cervellati and Sunde 2007; Clark 2005; Doepke 2004; Ehrlich and Kim 2005; Galor 2012; Lagerlof 2003; Strulik and Weisdorf 2014; Weisdorf 2004). This view has not been challenged even by economic historians with a strong background in historical demography. Thus, in a widely cited guide on the demographic transition for an economics audience, Guinnane (2011) reviewed the factors that might lead higher desired numbers of births pretransition, but he has been silent as to whether there was parity-specific control before the transition.

This economics literature will be convinced of an absence of parity-dependent control only by demonstrations to the contrary based on quasi-random interventions, which was not the method of the 1960s and 1970s historical demography study of natural fertility. In the following section, we review the traditional historical demography tests of natural fertility and the reasons for returning to this subject despite the earlier consensus.

We use the biological accident of twin births to confirm that in the pre-industrial Western European populations examined (including Québec, whose population derived mainly from France), there was no conscious attempt to control fertility. Families experiencing a twin birth ended up on average with 1 additional childbirth compared with those with only singleton births. In contrast in modern populations with fertility control, twins result in an increase in births within families that is significantly less than 1. However, given that families in the pre-industrial world with children had average numbers of births of 6 or more, if they had target numbers of births, they could adjust more easily to the biological accident of a twin birth than in a modern world, where the average number of births per family is only 2-–3.^2^ These pre-industrial families also often ended up, depending on relative twin and singleton death rates, with additional children surviving to age 14 and older than comparable families with a single birth at the same parity. There is no sign of any change in later fertility behavior in response to the accident of twining.

Our approach, using the random occurrence of twin births, has the advantage of being agnostic about the exact means that couples were employing to limit births: stopping, spacing, or some combination. If twinning induces earlier stopping, we will detect the effect. If twinning induces greater spacing between births, we will also detect the effect. We can test simply whether the accidental occurrence of an additional birth through twinning creates any behavioral response in families toward limiting fertility either through earlier stopping or increased spacing.

The twins test, however, will not detect deliberate pre-industrial fertility control in response to adverse economic conditions. Nevertheless, it would be surprising if families had the ability and inclination to reduce fertility but used that capability only in response to external economic shocks and not to the equally significant shock of having many surviving children to provide for.

## What We Know About Pretransition Fertility Control

Henry (1961:81) defined natural fertility as “fertility which exists or has existed in the absence of deliberate birth control.” In the natural fertility regime, fertility depends only on physiological and social factors affecting the level of fecundity. Henry identified 13 populations that he considered natural fertility regimes, although realized fertility varied considerably across these groups. Parity-dependent birth control in other populations was identified by observing a decline in fertility relative to natural fertility populations at older ages for women.

This raises an immediate logical issue about how we know whether even in the reference group, there is an absence of any parity-specific control. The natural fertility literature of the 1960s to 1980s did not specifically test whether fertility truly was uncontrolled in such populations. Fertility levels at any age, however, varied substantially across the 13 reference populations. These level variations were not seen as evidence of parity-specific fertility control. Control was evidenced only by deviations from the age pattern of natural fertility. The reference populations were assumed, without any formal tests, to practice no fertility control. The decline in fertility with maternal age was asserted to be completely a product of declining fecundity.

Coale (1971) introduced the parameters *M* and *m* as a way of formally characterizing fertility regimes and testing for the presence of parity-dependent birth control. *M* represents the average ratio of observed fertility relative to natural fertility at a given age, where natural fertility was initially represented by the Hutterite population, an early–twentieth century Anabaptist religious group that married early and prohibited contraception. *m* is the deviation of the observed age pattern of fertility from that of a natural fertility population, again represented by the Hutterites. *m*, alone, was the measure of parity-specific control. Thus,

If *m* = 0 the resultant schedule is simply a constant multiple at every age of “natural” fertility (represented by the Hutterite schedule); if *m* = 1 the schedule deviates from natural fertility to an extent that is the average degree of deviation of 43 schedules in the early 1960’s; if *m* is very large the schedule has very rapidly diminishing ratios of fertility relative to the Hutterite schedule as age increases. Only the second of the parameters (*m*) affects the age structure of fertility; the other (*M*) only helps determine the level of fertility. (Coale 1971:207)

Coale and Trussell (1974:185) presented model schedules of fertility designed to be “schedules encompassing the full range of human experience.” These model schedules are based on *M* and *m* parameters applied to natural fertility schedules. When does *m* indicate fertility control? Threshold levels were proposed as “*m* = 0*.*2 (very moderate control of fertility) and *m* = 0*.*4 (quite moderate control of fertility)” (Coale and Trussell 1974:195). In other words, no matter what the level of fertility, natural fertility populations are characterized by a relatively invariant and convex age pattern of fertility. Coale and Trussell later showed that in the 10 well-documented (of the original 13) supposed natural fertility populations identified by Henry, the estimated value of *m* was between –0.152 and 0.236 (Coale and Trussell 1978:205, table 2).

Regarding the *M* and *m* parameters, Knodel and van de Walle (1979) concluded that application of this technique to the results of the many family reconstitution studies, as well as to official statistics when available, indicates that family limitation in Western Europe was either absent or quite minimal (perhaps limited only to special segments of society, such as social elites) prior to the onset of the long-term decline in marital fertility. When the index of family limitation can be computed prior to the secular decline in fertility, it is usually close to zero and unchanging . . . [T]he evidence does not suggest that family limitation was practiced at some moderate but constant level prior to the secular fall in marital fertility rates. Instead, its incidence seems to have been quite minimal and in many cases completely absent (p. 227).

Further, they argued, “Couples do not have target family sizes. They accept, in some cases reluctantly, as many children “as God sends” (Knodel and van de Walle:235). Similarly, the Cambridge Group for the History of Population and Social Structure concluded that for England before 1838, “small groups may have been practicing family limitation, but the reconstitution evidence suggests that such behaviour was restricted to a small minority of the population, if present at all” (Wrigley et al. 1997:461). Livi-Bacci (1986), however, detected evidence of parity-dependent control for some upper-class groups in Europe before 1850, using such measures as *m* and the mother’s age at last childbirth: aristocrats in France, Florence, and Milan; the bourgeoisie in Geneva; and families in Genoa.

However, the Coale-Trussell test has been criticized because it may detect only particular forms of parity-dependent birth control. Spacing might be systematically used in natural fertility populations to limit family size throughout the course of marriage (*slowing*) yet be undetectable from the Coale-Trussell *m* parameter. This was a possibility noted even by Knodel (1979:504). In later work, scholars looked for an effect of net parity on subsequent fertility, which allowed for a mix of spacing and stopping behavior. One such method was cohort parity analysis (CPA) (David and Sanderson 1988; David et al. 1988).

Further, both the *M* and *m* approach and CPA have been criticized regarding their ability to detect the presence of a minority of controllers within the population. Both involve significant assumptions about the nature of control or the characteristics of controllers versus noncontrollers. Thus Okun (1994:222), who tested the effects of these assumptions on the ability of these methods to detect control using simulations, summarized that, “neither *M* and *m* nor CPA can be used reliably to test alternative theories of the fertility transition when, as is often the case, the tests revolve around identification of a minority of controllers.” In particular, Coale and Trussell’s index *m* takes values very close to 0 (e.g., *<*0*.*2) in simulated populations in which as much as 40% of the population practices effective, parity-dependent control. In particular, values of *m <* 0*.*2 cannot justifiably be cited as evidence of the absence of significant fertility control (Okun 1994:221).

Okun’s simulations themselves have to employ a baseline fecundity that is estimated assuming, again, some populations observed with no parity-specific control. Thus, the methods for establishing the presence or absence of parity-dependent birth control employed by the European Fertility Project have significant weaknesses. Further, Coale and Trussell did not give any confidence intervals for their estimates of *m*. Given that these estimates of *m* are based on samples from modestly sized populations compared with another set of population samples, the possibilities are for substantial error components in the estimates of *m*. This further reduces the *M* and *m* method’s ability to detect with high confidence the absence of parity-dependent fertility control. Thus, this earlier literature is based on untested assumptions about the reference population and poorly detects the presence of substantial minorities of controllers.

Since the end of the European Fertility Project, the tendency in the published literature has been to challenge the conclusion that the pre-industrial regime in Europe was largely one of natural fertility. In particular, another method has emerged for estimating parity-dependent fertility control: hazards models for another birth are estimated controlling for economic circumstances, numbers of dependent children, net parity, and mother’s age. These models concentrate on spacing and the response of spacing to such factors as net parity. The published estimates from such models generally suggest significant pre-industrial fertility control in response to economic circumstances (Amialchuk and Dimitrova 2012; Bengtsson and Dribe 2006; Cinnirella et al. 2017, 2019; Dribe and Scalone 2010), to numbers of dependent children (Van Bavel 2004), or to net parity itself (Anderton and Bean 1985; Cinnirella et al. 2017, 2019; David and Mroz 1989a, b; Kolk 2011; Van Bavel and Kok 2010). For example, Van Bavel and Kok (2010:136–137) concluded, “the married couples in our Dutch study population were controlling their fertility by means of birth spacing before the onset of the fertility transition.” And Cinnirella et al. (2017:413), noted, “Our findings on the existence of parity-dependent as well as parity-independent birth spacing in England are consistent with the growing evidence that marital birth control was present in pre-transitional populations.” However Clark and Cummins (2019) showed that the Cinnirella et al. (2017) results were an artifact of the estimation methods with impossible implications.

Thus, the debate on whether parity-dependent birth control existed in pretransitional populations is unresolved.

Another factor suggesting the possibility of parity-specific control in pretransition populations is substantial social class differences in gross fertility. Wealthier families in England marrying before 1780 had substantially more births within marriage than poorer ones, with shorter birth intervals and later stopping (Clark and Cummins 2015; Clark and Hamilton 2006). This difference within marriage was also linked to social status in England (Boberg-Fazlic et al. 2011; de la Croix et al. 2019). Similar patterns have been found in pretransition France (Cummins 2013, 2020). It is unclear what created this difference, but this again creates the possibility that deliberate parity-specific fertility control existed.

## Using Twins to Detect Parity-Dependent Birth Control

Twins have been estimated to represent about 1.8% to 2.7% of all births in pre-industrial European populations (0.9% to 1.9% of deliveries).^3^ Although twin births are more common among older women, they are largely a random event. There is only a modest tendency to repetition within the same family, with (as we show) little or no connection with economic and social status. With an average of 6 births per married woman (who had at least one birth), about 5% to 11% of families with children would experience a twin birth in the pre-industrial era.

Consider a population with no fertility control within marriage. In this case, whenever and however the marriage terminates, the expected number of births will be increased by 1 with a twin birth, assuming that the twin birth has no effect on the length of the subsequent birth interval. Also, the increase in the final number of births will be the same whatever the parity at the time of the twin birth.

**Fig. 1:**
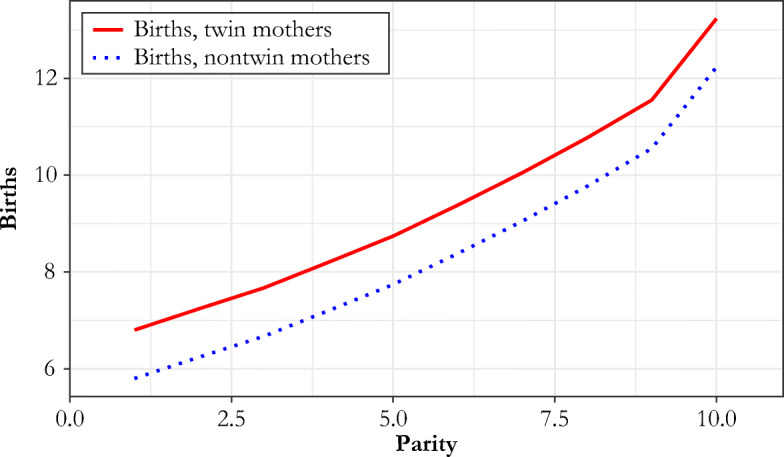
Expected effect of twins on total births

Figure 1 shows the expected effect of twins on total births by parity at the time of the twin birth, with parity-independent fertility. If we define net fertility as the number of children born to the family reaching age 14, then with uncontrolled fertility, the effect on net fertility will be less than 1 because of infant and child mortality. It also matters that twins showed a higher child death rate than singleton children (see upcoming Table 2). With uncontrolled fertility, the increase in the number of surviving children would be 2θ_*t*_ – θ_*s*_, where θ_*t*_ is the twin survival rate to age 14, and θ_*s*_ is the single child survival rate.^4^

In contrast, in populations where families control fertility and have a target number of children entering marriage, twin births will induce a more muted increase in completed family size. Suppose, as seems reasonable, the target is defined in terms of children reaching age 14. Call this number for family *i*, *N*_*i*_. Assume also the marriage lasts to the end of planned births. Then with only singleton births, the number of births needed to achieve this target will average *N*_*i*_ / θ_*s*_. With a twin birth, the number of births required to achieve the target becomes

$$ 2+\left(\frac{N_i-2{\uptheta}_t}{\uptheta_s}\right)=\frac{N_i}{\uptheta_s}+2\left(1-\frac{\uptheta_t}{\uptheta_s}\right). $$

As long as θ_*t*_ *>* 0*.*5θ_*s*_, the number of additional births required to achieve the target will be less than 1. If θ_*t*_ = θ*s*, then there will be no additional births.

However, if the twin birth is the last planned birth, it will add 1 additional birth (assuming θ_*t*_ *>* 0*.*5θ_*s*_). Because all marriages end with a last birth, the fraction of twin births that occur as the last planned birth will just be the inverse of average family size. In England, for example, for men whose first marriages took place 1730–1879, average family size for those with at least one child was 5.9. Thus, if each family had a planned target size, a twin birth would occur as the last planned birth 17% of the time. Suppose in general that the fraction of births that are the last planned birth is ϕ. For a marriage reaching completion of planned births, a twin birth will on average induce an increase in births of

$$ 2\left(1-\frac{\uptheta_t}{\uptheta_s}\right)\left(1-\upphi \right)+\upphi . $$

The number of additional children reaching age 14 induced by the twin birth will, however, be just ϕ(2θ_*t*_ − θ_*s*_) with controlled fertility.

To summarize, we expect the effect of a twin birth on the change in final family size to be the following, where ϕ is the fraction of births that are the last birth:

**Table:** 

All Births, No Parity-Dependent Control	1
All Births, Complete Parity-Dependent Control	2(1 − θ_*t*_ / θ_*s*_)(1 − ϕ) + ϕ
Surviving Births, No Parity-Dependent Control	2θ_*t*_ − θ_*s*_
Surviving Births, Complete Parity-Dependent Control	ϕ(2θ_*t*_ − θ_*s*_)

Also, with controlled family sizes, the effect of twin births in increasing total births and family size will be stronger the greater the parity at which a twin birth occurs because of the increased probability that the twin birth is the last birth planned in this family. Indeed, if the twin birth is not the last birth recorded for the family, then with control, it should have no effect on average net family size.

To give a sense of the magnitudes of the posited effects on births and net family size with and without families targeting fertility, consider the case of England for marriages 1730–1879, where ϕ = 0*.*17, θ_*s*_ = 0*.*65, and θ_*t*_ = 0*.*55*.* For uncontrolled fertility, births increase by an average of 1 with a twin birth, and net fertility increases by 0.44. For families with target net fertility and completion of that target, births increase by an average of 0.43 with a twin birth, and net fertility increases by an average of just 0.07 children.

To estimate the effect of twin births on family size, however, we have to control for the parity at which the birth occurs. The more births, the greater the chance of a twin birth. Thus, in terms of simply raw size, families with twin births will be larger. Twin births are also more common in older mothers. Thus, at a given parity, mothers giving birth to twins are, on average, slightly older than mothers of singletons. In the Families of England sample of marriages 1730–1879, this age difference implies that mothers of twin births at a given parity are 0.93 years older than for mothers of singletons. This will lead to mothers of twins having lower expected future fertility: it will bias the estimates downward. To have a truly comparable set of singleton births, we therefore have to also control for mother age at marriage. Thus, to test for the effect of twin births on total births, we postulate

1 $$ {NB}_{pk}={\upalpha}_b DTWIN+\sum {\upbeta}_j{DPARITY}_j+\sum {\updelta}_l DM{AGE}_l+\upvarepsilon, $$

where *NB*_*pk*_ is the total number of births in a family with a birth observed at parity *p* and mother age *k*, *DTWIN* is an indicator for that birth being a twin, the *DPARITY*_*j*_ are indicators that are 1 at parity *p* and 0 otherwise, and the *DMAGE*_*l*_ are indicators that are 1 at the mother’s age *k* and 0 otherwise. For populations with no control of fertility, α_*b*_ will average 1. Where there is complete fertility control, α_*b*_ will vary depending on the relative child mortality rates of twins and singletons and the average number of births per family.^5^

Similarly, if we look at completed family size NS, we would estimate

2 $$ {NS}_{pk}={\upalpha}_{\mathrm{c}} DTWIN+\sum {\upgamma}_j{DPARITY}_j+\sum {\upeta}_l{DMAGE}_l+\upvarepsilon . $$

For pre-industrial populations with no fertility control, α_*c*_ will vary but will often exceed 0 by significant amounts. Where there is complete fertility control but pre-industrial average family sizes, α_*c*_ will average only 0.0–0.10. Although easy to implement, these estimates are parametric estimates that assume no interaction effects between mother’s age and parity. An alternative completely nonparametric estimate of the effects of twins uses simply average family size for a mother of age *k* at parity *p* when the birth is a singleton versus average family size when the birth is a twin. This nonparametric estimate, however, does not use all the data because some cells in the mother age–parity matrix contain no twin births. We check our estimates in all cases using this nonparametric alternative. If we define a set of indicator variables for each combination of parity and mother age, *D*(*PARITY*_*j*_*, MAGE*_*l*_) that has value 1 at parity *p* and mother age *k*, then the estimating equations will be

3 $$ {NB}_{pk}={\upalpha}_b DTWIN+\sum {\upbeta}_{jl}D\left({PARITY}_j,{DMAGE}_l\right)+\upvarepsilon . $$

4 $$ {NS}_{pk}={\upalpha}_c DTWIN+\sum {\upgamma}_{jl}D\left({PARITY}_j,{DMAGE}_l\right)+\upvarepsilon . $$

With natural fertility, a twin birth will add one child to the family regardless of the parity at which the twinning occurs. If all families in a population are controllers with target family sizes, the average effect of a twin birth on total births and on completed family size will be greater the higher the parity at which a birth occurs. Pison and Couvert (2004) found that in France for fertility surveys 1975–1999, the chance that a mother aged 25–29 who gave birth to a singleton at parity 0 gave birth to a second child was .77. Thus, a twin at parity 0 induced an increase in family size of, on average, 0.23 children. However, the chance of a mother with singleton births at parity 0 and parity 1 would have a third child was 0.52. Thus, a twin at parity 1 induced a greater increase in family size of 0.48 (Pison and Couvert 2004: figs. 10–11). With a population that is a mix of natural fertility couples and controllers, however, the relationship between parity and the magnitude of the effect would be complicated by the greater share of high-parity births from natural fertility couples. We can test whether the pattern of additional births with parity is consistent with an entire population of noncontrollers by estimating the following equations:

5 $$ {NB}_{pk}={\upalpha}_b DTWIN+{\uplambda}_b DTWIN\cdotp PARITY+\sum {\upbeta}_j{DPARITY}_j+\sum {\updelta}_l DM{AGE}_l+\upvarepsilon, $$

6 $$ {NS}_{pk}={\upalpha}_c DTWIN+{\uplambda}_c DTWIN\cdotp PARITY+\sum {\upgamma}_j{DPARITY}_j+\sum {\upeta}_l DM{AGE}_l+\upvarepsilon, $$

where *PARITY* is the parity of the twin birth. With no fertility control, λ_*b*_ and λ_*c*_ should be 0.

Another way to check for a behavior response to the accident of twinning is by looking at the length of the birth interval following a twin birth compared with a singleton at a given parity and mother age. If twin births add exactly one birth to the total number of births in a family, then this interval should be the same following a twin birth as following a singleton.^6^ If there is a behavioral response, then the interval will potentially lengthen as families seek to reduce future births, as would be predicted based on Van Bavel’s (2004) finding that the number of children under age 10 increases subsequent birth intervals. Thus, we estimate the parameter ω in the equation

7 $$ In{terval}_{pk}=\upomega DTWIN+\sum {\upbeta}_j{DPARITY}_j+\sum {\updelta}_l DM{AGE}_l+\upvarepsilon, $$

as a potential detector of behavioral responses to twinning.

## Data

The data for analysis are multiple, independently constructed family history databases: the Families of England (FOE) database for England, the Henry data for France, the CAMPOP data for England, and the Québec IMPQ (*l’Infrastructure intégrée des microdonnées historiques de la population québécoise*) database. Table 1 summarizes the data available from each of these sources by period. The latter three databases use the techniques of family reconstitution: “Life consists only of birth, marriage and death. If the dates . . . of each member of a family are known, the reconstitution of that family is complete” (Wrigley et al. 1997:13). Many people in these databases, however, have a baptism record but no burial record, or vice versa.

**Table 1:** Summary statistics for studies

Country	Number of Births	Number of Potential Twins	Potential Twin Rate	Number of Parents	Year Min.	Year Max.	Source
France	130,746	3,756	0.029	35,849	1600	1788	Henry
France	49,742	1,520	0.031	16,018	1789	1895	Henry
England	59,687	950	0.016	10,153	1730	1949	FOE
England	31,001	814	0.026	11,287	1900	1949	FOE
England	76,959	1,355	0.018	7,731	1539	1826	CAMPOP
Québec	374,082	8,780	0.023	52,725	1625	1835	IMPQ

*Note:* Years refer to observed births.

### Families of England (FOE) Database, England

The FOE database is a set of complete family genealogies for English families with births in the interval 1730–2007, comprising 296,489 individuals. Because this is a new database, we detail how it was constructed in the online appendix. We constructed two samples of twins from this database. The first sample was drawn from men whose first marriage occurred 1730–1879 and whose fertility record appears complete. The dates here were chosen as those for which marriages appear to have largely uncontrolled fertility, as measured by births per father. The date range for the twin births in this period is therefore 1730–1915.^7^ Of the near 60,000 births attributable to these fathers, 471 deliveries were identified as a pair of twins—a twinning rate of 1.6% for all births. The second comparison sample is of twin births 1900–1949 to men whose first marriage occurred in 1880 or later, a period when couples were clearly exerting some fertility control, with nearly 31,000 births and 406 pairs of twins (2.6% of births). The higher proportion of twin births in the early twentieth century is quite consistent with the history of twinning. National figures for twinning rates for England starting 1938 show a twinning rate of 2.5% 1938–1949 (Pison and D’Addato 2006).

Detecting twin births in historical data sources is not a trivial exercise. In particular, in England, where attendance in the established Church of England was not particularly strict in the eighteenth century and later, children were sometimes not baptized until years after their birth. Thus, the baptismal records contain cases where nontwins are baptized on the same day. The FOE data have the advantage that births were registered to within one-quarter of a year for 1837 and later. Also, children born in 1841 and later appear on census records, where if they are twins, they will be listed with the same age. Thus, for births after 1830, we have multiple other sources indicating whether they are truly twins. For the second period—births in 1900–1949—we know the mother’s name from the birth record in 1911–1939, which for rare names almost conclusively identifies twins in these years. For births in 1900–1911, we see both children if they survive in the 1911 census. Thus, the accuracy of twin attributions is high for 1900–1949, but we must rely on baptism records for births in 1745–1830. Where we have complete fertility records, however, we can see cases where a multiple baptism is preceded by a gap of more than three years in baptisms, and we do not include such potential nontwin births.

### Henry Data, France

The French data are the complete Enquête Louis Henry–led demographic survey of 41 rural French villages, 1670–1895.^8^ To allow mother’s dates of death to be observed, we look at twin births just in the interval 1670–1829. The period covered by the Henry data covers two periods: (1) 1670–1789, which traditionally was regarded as being a period of natural fertility; and (2) 1789–1829, when families were believed to be exercising some fertility control. The Henry data contain a field indicating whether a child was a twin. Because of the Catholic practice of baptizing children as soon as possible after birth, the detection of twins is reliable in the Henry database.

### CAMPOP Data, England

The CAMPOP data were assembled in the same way as the Henry data for 26 English rural parishes. It also has a field indicating whether a child was a twin. However, as noted earlier, twins are detected in the CAMPOP data only through the baptismal records. The baptismal records only sometimes explicitly note that children baptized on the same day are twins. We do not know how the creators of the CAMPOP data concluded that the children they identified as twins were indeed twins. We show shortly that we can test for the reliability of the twin designation using the same-gender ratio for the twins. On this test, the share of same-gender children is too low in the CAMPOP data, implying significant numbers of misidentified twins.

### IMPQ, Québec

The *Infrastructure intégrée des microdonnées historiques de la population du Québec* (IMPQ) is a set of family reconstitutions of the Catholic population of Québec using baptisms, burials, and marriages 1621–1849 (Dillon et al. 2018; IMPQ Project 2019; Project Balsac 2019; *Programme de recherche en démographie historique* 2019). Not all births are linked to death records. We consider only those born in the interval 1621–1835, interpreting the lack of a death record as survival until age 14. As mentioned earlier, Catholic doctrine strongly encouraged prompt baptisms, going as far as threatening delinquent parents with excommunication; thus there should not be any significant occurrence, as in the English baptismal data, of nontwins being baptized on the same day. To ensure that completed family are observed, we restrict the sample to families where the father was born in Québec, married before 1830, and all children have a known parish of birth in Québec.

The unit of observations could potentially be one of three things: births per mother, births per father, or births per marital union. For our purposes, the ideal measure is all births per mother or all births per father: if a marriage is terminated early by the death of one party, the other has the option to remarry to attain the desired family size in the presence of controlled fertility. We would also ideally use only mothers who reached age 40 or fathers who reached age 45 in order to observe close to complete reproductive intervals. However, in the Henry data, the CAMPOP data, and the Québec data, birth and death dates can be missing. In the main estimation tables, we therefore include all families except those in which the parents are known not to have attained age 40 for women and 45 for men in the Henry and CAMPOP, and age 45 for men in the FOE and Québec.

For the Henry and CAMPOP data sets, we take the unit of observation as marital unions simply because of how the data were constructed. For the FOE data, which were constructed around fathers with rare surnames, the unit is total births per father. Cases in which first wives died before age 40 are included given that men had the option of remarrying. For the Québec data, we can measure either total births per father, mother, or marital union. Here we chose to use births per father because twin births in Québec were associated with a significant increase in observed maternal mortality in childbirth (from 1.3 to 4.0 per 100 births). If we instead use fathers, there is no issue of twinning-induced parent mortality. If a mother died in childbirth, the father would often remarry. Some fathers will have died before their wives reach the end of their reproductive careers, but this effect will be found equally among twinning and nontwinning families.

We can test the accuracy of our twin attributions by looking at the gender composition of twins. Two children who are not twins have a roughly 50% chance of being of the same gender.^9^ Monozygotic (MZ) twins, of course, have the same gender. They show a constant rate across a wide variety of societies at roughly 0.7% to 0.9% of children (Pison and Couvert 2004:769). Twining rates for dizygotic (DZ) twins vary substantially across time and across populations; however, for each twining rate is an implied same-gender ratio among twins. We can test what fraction of actual twining events we are correctly identifying in our data, ϕ, because the fraction of the putative twins having the same gender will be

8 $$ {R}_{SS}=0.5+\frac{0.4}{R_t}\upphi, $$

where *R*_*SS*_ is the same sex ratio, and *R*_*t*_ is the observed twinning rate per 100 births. If we miss many true twining events and instead misattribute singleton births, then the gender ratio will be closer to 0.5 than predicted by this formula when ϕ = 1*.* In general, as Table 2 shows, the French and the Québec data have sex ratios consistent with complete detection of true twinning events. For the FOE sample for twins born in marriages before 1880, the fraction that were same sex was .64, which is less than an expected rate of .72. So, for the FOE data, there is some evidence that not all twins are being detected and some nontwins are included. For the CAMPOP data, however, the same-sex ratio for their identified twins is only .59, compared with an expected rate of .70, implying that many twins were not detected. Table 2 shows actual and expected same-gender shares for the various twin samples. This test suggests that the Henry and IMPQ data are of the best quality, followed by FOE, but with CAMPOP possibly including many nontwins in their twin attributions.

**Table 2:** Twin parameters

Sample	Survival Rate, Nontwins	Survival Rate, Twins	Average Births per Marriage	Same-Sex Ratio, Twins	Same-Sex Ratio, Expected
France, pre-1789	0.71	0.47	5.31	0.64	0.62
France, post-1789	0.70	0.41	4.66	0.68	0.61
England, CAMPOP	0.70	0.46	4.66	0.59	0.70
England, 1780–1879	0.65	0.55	5.96	0.64	0.72
England, 1900–1949	0.91	0.72	3.37	0.55	0.63
Québec	0.71	0.52	5.64	0.68	0.65

The effects of attributing twins when the children in a family are actually singletons will be to bias the estimated coefficients on *DTWIN* toward 0. Thus, mistakes here in the data will bias us toward finding evidence of fertility control.

Table 1 reports the summary statistics for the studies used: how many births, how many potential twin births, and the years covered. Table 2 reports the diagnostic parameters for the twin samples: the survival rate to age 14 of singleton births and twins, the average number of births per family, the same-gender ratio for the putative twin births, and the expected same-gender ratio.

**Fig. 2:**
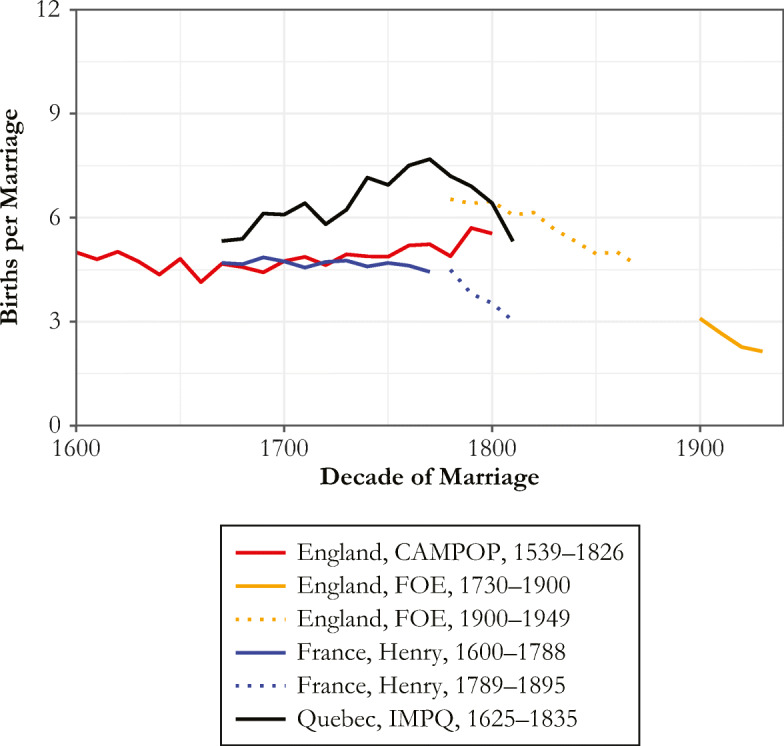
Average births, by sample and decade

Figure 2 reports the mean number of births, for women surviving to at least 40, for each of the samples. Two of the six samples—France for marriages 1790–1829 and England 1900–1949—show signs of fertility control in having substantially lower numbers of births per marriage or per father.

Twinning is mostly uncorrelated with the observable social characteristics of families. Table 3 shows the coefficient for each of a variety of parent characteristics regressed singly on an indicator for whether a birth is a twin: mother’s age (in years); parity; the first to second birth interval as an indicator of fecundity (which correlates with total births); mother’s literacy; and father’s literacy, education, occupational status, and wealth. Mother’s age correlates significantly with twinning rates in all except the Henry data.^10^ For example, for marriages 1730–1879 in the FOE database, the implied twin birth rate is 0.98% at mother’s age 20 but 2.07% at age 40. Parity is always significantly correlated with twinning rates but will be highly correlated with mother’s age, so part of the parity effect will be an age effect. However, Pison and Couvert (2004: fig. 4) showed that even controlling for mother’s age, there is a positive parity effect. Pison and Couvert (2004:770) also reported, “These differences . . . have been interpreted as resulting from a physiological phenomenon (Henry, 1975), though the mechanism is unknown.” The correlation with age of the mother and parity is not a problem because we control for both of these in the estimations.

**Table 3:** Twinning correlates

Variable	France, Henry	England, CAMPOP	England, FOE	Québec, IMPQ
Mother’s Age	–0.00005	0.0005**	0.0009**	0.0004**
	(0.003)	(0.0001)	(0.0001)	(0.00003)
Parity	0.03**	0.002**	0.004**	0.0006**
	(0.002)	(0.0002)	(0.0002)	(0.00005)
First to Second Birth Interval	0.002	–0.002*	–0.002	0.00002
	(0.004)	(0.001)	(0.002)	(0.00004)
Literate Father	0.003	0.0001		0.0003
	(0.019)	(0.002)		(0.0006)
Literate Mother	–0.0005	0.002		0.0008
	(0.026)	(0.002)		(0.0007)
ln(Wealth), Father			0.0001	
			(0.0003)	
Occupational Rank, Father			–0.000002	–0.005
			(0.00004)	(0.003)
Educated Father			–0.004	
			(0.003)	

*Note:* Standard errors are shown in parentheses.

*p < .05; **p < .01

A more important issue is whether twinning correlates with fecundity. As shown in Table 3, our proxy for fecundity—the first to second birth interval—is never significantly associated with higher twinning rates. Given that Pison and Couvert (2004:785) reported that, “The most fecund couples have a greater propensity to bear twins,” this seems surprising. However, the basis of Pison and Couvert’s assertion is an association between the first-birth interval and the chance of a twin birth as the first birth. For first-birth intervals between 10 months and 36+ months, there is no association between the length of the interval and the chance of a twin birth, either in early twentieth century France or in the Henry data. The positive association between a short interval and twinning appears only for first-birth intervals of 8–9 months (but not for even shorter first-birth intervals). However, in the modern United States, the average pregnancy length for twin births is 35 weeks. Thus, an unusual proportion of twin births will occur in the 8–9 months category (Pison and Couvert 2004:787, fig. 15; 792, appendix table 2). Therefore, the Pison and Couvert data are perfectly compatible with our finding of no positive association between fecundity and twinning rates.

Pison and Couvert (2004:770) also reported that DZ twins “have a tendency to be repeated among the same women.” A test for whether some couples have a higher tendency to produce twin births comes from looking at the incidence of multiple twin births for a given father. In the FOE data with 473 twin deliveries for marriages before 1880, there are 18 cases of two such deliveries in a family and 1 case of three sets of twins. If we randomly allocate twin deliveries across the observed deliveries per father at the observed twining frequency, we find 11 cases of two such twin deliveries in a family (with a standard error of 3), implying only a slight tendency in some couples toward twin births. However, any distorting effects this would have on the estimation will be small. We show shortly with the FOE data for 1730–1879 that the 473 families produce 473 extra births. The familial association of twinning accounts for 9 of these extra births. Thus, the familial association will bias upward the estimated effect of twinning on births by 0.019. The simulation also implies that from the perspective of couples, twins represent overwhelmingly random and unpredictable events.

## Results

Table 4 summarizes the estimates of α_*b*_ the effect of a twin birth on total births for the six population samples we have for Eq. (1), which controls for mother age and parity. In three of the four pre-industrial population samples—FOE, Henry, and Québec—the estimate of α_*b*_ is very close to the value 1 predicted by natural fertility. In the case of Québec, the standard error of this estimate is only 0.05, so this is a very precise estimate. In these cases, the estimate of α_*b*_ is also significantly above the value that would be predicted from having control and a target family size. In the case of the CAMPOP data, however, the estimate of α_*b*_ is closer to that produced by control than by natural fertility, although it is not statistically different from 1 at the 5% level. However, as shown in Table 2, the CAMPOP twins show a greater deviation from the predicted same-gender ratio for twins than any other sample. This deviation implies that singleton children are misclassified as twins and will bias the estimate of α_*b*_ toward 0.

**Table 4:** Twin effect, births

Sample	Expected α, No Control	Expected α, Control	α	SE	N
Pre-Fertility Decline					
France, pre-1789	1	0.73^a^	1.02	0.07	65,722
England, CAMPOP	1^a^	0.75	0.83	0.09	76,239
England, pre-1880	1	0.43^a^	0.99	0.12	55,590
Québec	1	0.61^a^	1.03	0.05	324,202
Post-Fertility Decline					
France, post-1800	1	0.87	0.89	0.12	18,454
England, 1900–1949	1^a^	0.60	0.72	0.10	27,399

^a^Hypothesized coefficient is rejected at *p* = .05.

We turn now to the populations exercising at least some fertility control: England 1900–1949 and France 1800–1829. For England, α_*b*_ falls statistically significantly below 1 at the 1% level but is not statistically different from the value predicted for families with a target family size. For France post-1800, the expected α_*b*_ with fertility control, because of the low survival rate of twins, is close to 1 at 0.87. The estimated α_*b*_ at 0.89 is close to that predicted by fertility control, but the standard error is large enough that the estimated coefficient is compatible with either control or its absence. The estimates for births are consistent with the earlier populations having no control of fertility and the later ones having at least some controllers.

What about the possibility that even though the majority of pre-industrial families were not exercising any parity-dependent control, a significant minority were exercising such behavior? Given the standard errors in Table 4, we can be 95% confident that no more than 3% of the Québec population had a target family size, but for both England and France pre-1789, given smaller sample sizes, at the 95% confidence interval we can conclude that at most only 35% of families were exercising control. We can state, however, that in France pre-1789, with 75% probability, less than 10% of families had a target family size they controlled. For England pre-1880, with 68% probability, less than 10% of families were exercising control (and for Québec, we can conclude that with 98% probability). Thus, our study contributes evidence that most likely almost no families in France pre-1789, England pre-1880, and Québec pre-1835 exercised any targeting behavior with respect to fertility.

Although the samples used here may not allow us to reject any pre-industrial targeting behavior at the 95% confidence limit in France and England, much larger bodies of data are likely to become available. Although in England, our sample includes 55,533 births for marriages before 1880, there were 28,700,565 English births 1838–1880. For those with rarer surnames, 10% or more, twins can be identified with high reliability from the birth register. It will be possible using curated family trees from Ancestry.com and other genealogical services to obtain sample sizes that will conclusively establish whether there was any parity-dependent control of fertility before 1880 (recent studies have exploited such curated data to investigate the inheritance of longevity; see, e.g., Ruby et al. (2018) and Kaplanis et al. (2018)). Similarly, research teams are at work extending the information on births and deaths in Québec from 1850 to 1916. The sample size for pre-industrial Québec, for which we already have a good estimate of the likely share of controllers, is likely to be two to three times as large within a year.

**Fig. 3:**
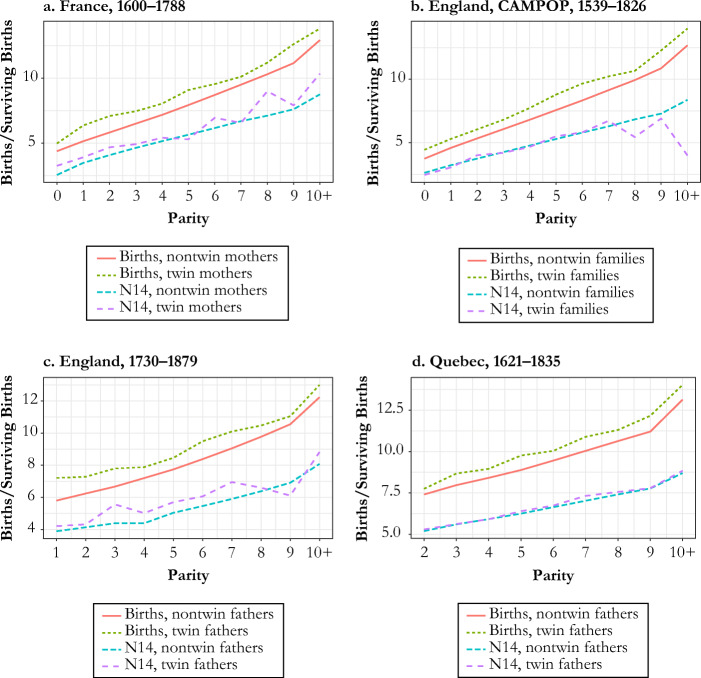
Observed births by parity at twin birth

If we instead estimate α_*b*_ nonparametrically from Eq. (3), where interactive effects between parity and mother’s age are allowed, we find a very similar set of results as in Table 4. Figure 3 illustrates the pattern of total births and completed family size (measured here as the number of children attaining age 14) at each parity for twin births versus singleton deliveries for France 1670–1789 (Henry), England 1538–1826 (CAMPOP), England 1730–1879 (FOE), and Québec 1621–1835 (IMPQ). In each case, there is a clear sign that the increase in total births created by twinning has the same magnitude independent of the parity at which the birth occurs. Again, this is consistent with the whole population exercising natural fertility.

Table 5 shows the formally estimated coefficients for λ_*b*_ and λ_*c*_, the effect of parity at the twin birth on total births and completed family size, from Eqs. (5) and (6) for the supposed natural fertility populations. These coefficients are all not distinguishable from 0 for the populations with hypothesized natural fertility, which is a sign that the increase in family size from twinning was as strong for the first birth being a twin as for births at high parities and is entirely consistent with a whole population of noncontrollers.

**Table 5:** The effect of twinning as function of parity

Sample	γ_*b*_	SE	*N*	γ_*c*_	SE	*N*
France, pre-1789	–0.03	0.03	65,722	0.05	0.04	24,609
England, CAMPOP	0.06	0.04	76,239	0.03	0.04	76,239
England, pre-1880	–0.09	0.05	55,590	-0.04	0.05	52,706
Québec	–0.01	0.10	324,202	-0.02	0.09	324,202

*Note:* Parity coefficient is from OLS estimation of Eqs. (7) and (8).

Because of the higher infant mortality rates for twins, the effects of twinning on completed family sizes with natural fertility are smaller than on births. Table 6 summarizes the estimates of α_*c*_ in Eq. (2), which shows the effects of twins on numbers of children reaching age 14 controlling for parity and mother’s age. For all the natural fertility populations with no parity-specific control, the number of surviving children should increase by 0.22–0.44 as a result of twinning; with family size targets and control, the increase would be only 0.04–0.07. The empirical estimates confirm a rise in net fertility significantly above what would be expected with target family sizes in England (FOE), France, and Québec. The only sample in which net fertility does not increase is CAMPOP. In the other three cases, the rise is as large, or even larger, than would be predicted from twin versus singleton survival rates.^11^ Thus the evidence for net fertility in England (FOE) implies with 95% confidence that no families were operating with target family sizes.

**Table 6:** Twin effect, surviving children

Sample	Expected α, No Control	Expected α, Control	α	SE	*N*
Pre-Fertility Decline					
France, pre-1789	0.24	0.04^a^	0.37	0.11	24,609
England, CAMPOP	0.22^a^	0.05	0.00	0.08	76,239
England, pre-1880	0.45	0.08^a^	0.70	0.13	52,706
Québec	0.33	0.05^a^	0.24	0.05	324,202
Post-Fertility Decline					
France, post-1800	0.12	0.03^a^	0.37	0.15	8,132
England, 1900–1949	0.52	0.15^a^	0.36	0.10	27,288

^*a*^Hypothesized coefficient is rejected at *p* = *.*05.

Table 7 shows, both for births and for surviving children, the 5% confidence intervals for the proportion of the population that potentially had target family sizes in the three populations for which we believe the twins are well identified.^12^ Two findings are noteworthy. First, in no case can we be confident at the 5% level that these pretransition populations had any controllers. Second, we can be confident at the 5% level that no more than 25% of the French pre-1789 might have been controllers, that none of the English pre-1880 might have been controllers, and that no more than 5% of the Québecois pre-1850 might have been controllers. For the two populations controlling fertility, the rise in net fertility from twinning is again greater than would be predicted from everyone controlling, which can be explained by these populations containing a mix of families, some with fertility controls and some without.

**Table 7:** 5% confidence intervals, proportion potentially controlling in sample populations

	Births	Surviving Children
France, pre-1789	.0–.35	.0–.25
England, pre-1880	.0–.36	.0–.0
Québec, pre-1850	.0–.05	.0–.52

*Notes:* The table shows confidence intervals for a one-sided test on the potential fraction of controllers.

We also estimate for the pretransition populations ω in Eq. (7) to test whether there was any lengthening or shortening of the birth interval immediately following a twin birth compared with singleton births at the same parity and mother’s age. Parity-dependent birth control could take the form of longer spacing after twin births. In all cases, the post-twins birth interval does not differ significantly from the interval after a singleton birth, which is consistent with the evidence of no behavioral response to twinning. For example, for Québec, which has the most abundant data, the point estimate of the effect of a twin birth is that it shortens the following birth interval by five days, with a standard error of nine days.

## Conclusion

There is good evidence that at least in some Western European and Western European–derived populations—England, France, and French Québec— there was a period where families exercised no parity-dependent fertility control within marriage, for at least the great majority of the population. The biological accident of twinning produced no behavioral response. A family that had a twin birth ended up with one extra child born, at whatever parity the twinning occurred. Depending on singleton and twin survival rates, it also generally saw some fractional increase in completed family size. Families were not attempting parity-dependent fertility control within marriage, even in England as late as the period of the Industrial Revolution, 1780–1879. Interestingly, this was an era in which there were already significant investments in education and training even for poorer English families. Among children born in 1840–1860 in the FOE database, 31% were at school or in training at ages 14–16 (and only 54% at work). Yet there was no sign that parents were limiting births within marriage to control such expenses.

Clark and Cummins (2019) argued that the finding of Cinnirella et al. (2017) of substantial parity-dependent birth control in England 1538–1850 was just an unfortunate artifact of the estimation method used. The absence of any behavioral response to twinning in England before 1879 reinforces the conclusion that parity-dependent spacing was also absent. The increase in parity induced by a twin birth would on the basis of the Cinnirella et al. (2017) estimates induce much longer spacings of subsequent births. That longer spacing would lead to little or no increase in total births from twinning. Yet, even within the CAMPOP data they employed, twining leads to a significant increase in total births.

The findings with the Henry data for France pre-1789 also cast doubt on the earlier claim of David and Mroz (1989a, b) to have found similar evidence for control through birth spacing in France 1749–1789.

With enough data, the nonresponse to twinning implies that twins can be used as an instrument for family size, as an exogenous source of variation in family size. However, the variance in size induced by twinning is a small component of the overall variance, so there would have to be enormous amounts of data to estimate with any precision the coefficients linking child outcomes to family size, especially if the relevant measure is completed family size as opposed to births. More promisingly, the response to twins—by showing that families were not choosing family size—suggests that in these populations, we can consider all the variation in family size as exogenous. In particular, in England, for marriages in the years 1780–1879, fertility was uncorrelated with family social status. Average completed family size was the same on average for the poorest as for the richest families. Thus in this period in England, we can get very simple estimates of the effect of family size on child outcomes given that the variation in family size is exogenous to the social status of the family and not a choice made by parents. In another paper, Clark and Cummins (2016) estimated these effects of child quantity on child quality. Although the quantity effect generally produces a statistically significant negative effect on child quality, the effect is very small in terms of magnitude (Clark and Cummins 2015, 2016). In the Québec sample, the simple estimates might be biased because fertility appears to be negatively correlated with social status. However, this sample is large enough to use twins as an instrument. Again, we find that child quantity has a statistically significant yet very small negative effect on child quality.

The lack of any fertility response to twinning by families in England throughout the years 1780–1879 is also interesting in light of recent theories of the Industrial Revolution. By 1780, the rate of technological advance had clearly increased significantly from that of the previous six centuries in England. Technological advance has been attributed to a democratization in England of the ideas of the Enlightenment, an intellectual movement that emphasized rationality, experiment, and embrace of novel theories of both science and society (see, e.g., Mokyr 2010). Although the originators were an elite group of philosophers and scientists, the claim is that in England by the late eighteenth century, these ideas had filtered down through lectures, demonstrations, and popular writings to the mechanics and artisans, whose many small-scale innovations underpinned the Industrial Revolution. The Industrial Revolution was mostly a product of a new way of thinking. Thus, “Economic change in all periods depends, more than most economists think, on what people believe” (Mokyr 2010:1). If the foundation of the Industrial Revolution was indeed new, more instrumental ways of thinking about the world, it is puzzling that this instrumentality did not also induce parity-dependent fertility control within marriage long before 1880.

## Electronic supplementary material

ESM 1 (PDF 174 kb)

## Data Availability

The datasets generated and/or analyzed during the current study are available from the corresponding author on reasonable request.

## Footnotes

1 See also Wrigley et al. (1997), who found support for the natural fertility interpretation in pre-industrial England.

2 For example, Braakmann and Wildman (2014: table 6) found that in a sample of 17,862 British women not receiving fertility treatments who had a birth in 2000–2001, six years later the average total number of births to those who had a multiple birth was only 0.66 greater than for those who had a singleton birth (standard error = 0.103).

3 Pison and Couvert (2004: figure 1) reported such a rate for France 1700–1989, based on the Henry data.

4 This number will be negative if twin survival rates are lower than singleton survival rates.

5 There are cases with multiple twins in one family. These are included in the estimate: a twin at a later parity will increase *NB* by the same amount regardless of whether the current birth is a twin and thus will not affect the estimate of α_*b*_.

6 Because infant mortality affects this interval, twinning could change the interval even without any behavioral response. But the chance of at least one surviving child after a twin birth will not be too dissimilar from the chance of a surviving child after a singleton birth. Twins have higher infant death rates, but there are two of them, so the chance of no surviving children a year after a twin birth is the square of the twin infant death rate.

7 We counted births for all marriages for these men, which explains why a man first marrying before 1880 could have twins in 1915.

8 The summary papers of the Enquête Henry are Henry (1972), Henry and Houdaille (1973), Houdaille (1976), and Henry (1978). A summary of all studies using the Henry data (before 1997) is listed in Renard (1997), and detailed discussion of the database can be found in Séguy and Méric (1997), Séguy (1999), Séguy and le Sager (1999), Séguy et al. (2001). See also Cummins (2013) for a recent analysis of the Henry data for fertility and wealth patterns during the fertility decline.

9 We assume in this calculation the same number of males as females at birth. In practice, the ratio is about 1.05 boys per girl. However, this means that two nontwin children will share gender 50.03% of the time compared with 50%, so the bias created by the simpler 50:50 assumption is very small.

10 In the Henry data, only a small proportion of mothers have birth dates listed, so the standard error of the estimated mother age coefficient is very large. Thus, we cannot rule out a substantial mother age effect also in this population.

11 For FOE, the rise in net fertility at 0.70 is significantly greater than the 0.44 that would be predicted with natural fertility, although only at the 5% level of statistical significance.

12 Because the question of interest is what proportion of the population at maximum could be families with target family sizes, we use a one-sided test.
