# Supplementary material for: Twins Support the Absence of Parity-Dependent Fertility Control in Pretransition Populations
Source: Demography. 2020 Jul 17;57(4):1571–95. doi: 10.1007/s13524-020-00898-0 (PMC7441081; doi:10.1007/s13524-020-00898-0)
Supplement: Supplementary file 1 — (PDF 174 kb) [file 13524_2020_898_MOESM1_ESM.pdf]

Online Appendix

Families of England Database

The Families of England database aims to construct a complete genealogy of a representative set of English families from 1730 to 201, a period of 9 generations, using public data sources. The database has been constructed primarily to examine the process of social mobility over multiple generations. But it contains substantial amounts of information also on geographic mobility, fertility, and mortality.

The database currently contains 296,494 individuals. The database is still a work under construction. The intergenerational linkages for these individuals are substantially complete for those born before 1930, but for those born later there is more work to be done on establishing these links. Currently there are 184,902 children linked with a father. There are 114,716 children of 27,515 fathers where the complete fertility history of the father is known. However there is substantial ongoing work on establishing occupations, educational status, dwelling values, and wealth at death for each individual. We expect to add considerably more data on all the social outcome variables.

To enable high linkage rate with the sources we have we adopted the strategy of following families with rare surnames, and follow descent in those families along the male line. The vagaries of English spelling, and the varied ethnic background of the population in different parts of England, ensures that a substantial minority of the English population, even in 1800, held surnames that were shared with modest numbers of other individuals. To ensure that there is no bias in this procedure we will also link many of the daughters to their husbands, and wives to their fathers, to check that mobility and other characteristics along the female line have the same character as with the male line. Using such rare surnames we can achieve very high linkage rates between parents and children.

For men born 1850-1949, and living to reproductive age, the linkage rate to a father for those born with one of the target surnames is greater than 90 percent. Typical linkage rates for historical intergenerational databases, using all surnames, at least in the US, are only around 20%.[17]. These linkages are also of high reliability in the years 1800-1930, since there are multiple sources in many cases identifying parents - censuses, birth records, marriage records, passenger lists - and there are few alternative candidates who can get confused with the target individual. Thus for a sample of 7,626 recorded rare surname births 1860-1879, we identify a father or mother for 88%[18]. The reasons for failing to find at least one parent in the other 12% of cases are various. In some cases the name likely was misspelled in the birth record, and the person does not belong in the surname lineages used to form the sample. Of those not linked 60% show no further appearance in any record after their birth under the birth name. Likely in most of these cases the name is just misspelled on the birth register. In others the child dies before appearing in a census, or their father dies, or they are living with grandparents in the census, or the family emigrates[19]. Thus one third of those born not linked to a parent died before age 10. Again, in contrast, historical intergenerational databases in the US using the general population are claimed to mismatch one third of individuals to their parents (Bailey et al., 2017). A reflection of the likely high success rate in making linkages is the observed intergenerational correlation of occupational status. This is 0.7, which is much higher than that observed in other census based historical linked samples.

Though the numbers of recorded births for men and women is similar, and the match rate to fathers for the births is also similar by gender, the final dataset of family size by father is missing at

[17] Long and Ferrie, 2018, for example, link only 20% of adult sons to their fathers in England between 1851 and 1881.

[18] In some cases, where the child is illegitimate, only the mother is listed on birth records.

[19] We could identify the father by getting the birth certificate, but this is prohibitively costly.

least 12-14% of girls. This is because children in families can also be identified from the existence of a death record, or from their presence in a census or other record, where the birth was not recorded under the correct family surname. But adult women will only appear in a death or census record if they remain unmarried. Thus more sons are identified from such records, absent the birth record. The absence of these women should be neutral, however, between twin and non-twin families.

To ensure a representative sample of people in each generation we have followed the strategy of including in the database all individuals bearing one of the target surnames whenever there is a birth, death or marriage record under that surname. We also try and follow the lineages of those who emigrate from England, typically to Canada, Australia, the USA, and New Zealand.

The genealogical linkages have been established in two ways. For a substantial subset of the data, 67,305 individuals we constructed the genealogical links ourselves. The other 229,189 individuals are from genealogies constructed by members of the Guild of One-Name Studies, a society devoted to studying the history and genealogy of rare surnames. The use of these Guild genealogies raises issues of selectivity, since it is more likely that a rare surname will be included in a Guild study if there is a current bearer of higher social status. But we do extensive checks on the representativeness of these Guild contributed surnames, and find that at least for the 19th century they have average social status.

In both our reconstructions and those of the Guild genealogies the familial linkages - assigning fathers, mothers, and spouses - are established using a wide range of evidence. For England there are census records 1841, 1851, 1861, 1871, 1881, 1891, 1901, 1911. There is the Population Register of 1939. There is the register of births, deaths and marriages 1837-2005. The birth register 1912-2005 gives the surname of the mother. There are selective parish registers of births and marriages 1730-1930. There are probate records nationally, 1858-2018, and for the Canterbury and York Ecclesiastical courts 1750-1858. There are passenger lists for those leaving the UK 1890-1960, and for those entering the UK 1878-1960. There are Electoral Registers 1900-2012.

In recalcitrant cases in England we can, at cost, order the actual birth certificate which list the father and mother, or marriage certificate which lists marriage partners, their occupations and those of the fathers. We plan on doing this for a select sample of people marrying around 1990, so that we can get their occupational status, where they would typically be born circa 1960, as well as the occupational status of their fathers born circa 1930.

It is possible in many cases to check proposed familial linkages against genealogies uploaded by ancestry.com members. These genealogies are not always reliable. But the better ones cite source documents which can be inspected to see if the link is sound.

Ancestry.com records the age at death of many migrants from the England to Canada, Australia, NZ and USA. For Australia the voting rolls 1903-1983 give occupations. For the US the censuses 1850-1940 record occupations. Canada and New Zealand also have some occupational information from voting rolls. However, wealth at death is generally not available for migrants outside England and Ireland.

The social status indicators we have are age at death, wealth at death, schooling, occupation, location, and first names of children.

Wealth at Death: For England and Wales the Principle Probate Registry records whether someone was probated, and the value of their estate for all deaths in England 1858-2018. This information is the most comprehensive and unusual outcome result that we have for this database. The probate information is searchable at https://probatesearch.service.gov.uk/#wills. However, the estate values 1996-2018 are now obtainable only at cost of 10 pounds per person.

Schooling and Training: The censuses of 1851-1911, and population register of 1939, record

whether anyone aged 10-19 is still attending a school, which gives us a measure of education for the earlier years. From the previous NSF project we have a database of all students who attended Oxford or Cambridge, 1750-2015. But this constitutes only 1-2% of each cohort. Complete records are available for attendees at the Royal Military Academy Woolwich (1790-1839) and Royal Military College Sandhurst (1800-1946). Complete records are available for Masters and Mates Certificates, 1850-1927, UK Medical Registers, 1859-2015, UK, Civil Engineer Lists, 1818-1930, UK, Electrical Engineer Lists, 1871-1930, UK, Mechanical Engineer Records, 1847-1930, UK, Articles of Clerkship, 1756-1874. From all these measures we can construct indices of educational attainment for people in the database born before 1900.

Occupation Status: The censuses of 1851-1911, and the Population Register of 1939 record occupations, so we can estimate adult occupations for the cohorts born 1920 and before. Passenger lists give occupations for international travelers up to 1960. Birth certificates record the occupation of father's, and from 1995 on that of mothers also. Marriage certificates record the occupations of husband and wife, and of fathers. So for a select sample we can estimate occupations for people born up to around 1980.

Dwelling Value: From the electoral census of 1999-2012 we have the address where adults were living in 1999-2012, from which we can infer using the Land Registry the property value in 2017. This gives an indirect measure of family income.

Children's First Names: Children's first names are a good proxy for family social status in modern generations. Using records of Oxbridge attendance and property values we can assign status measures to parents based on their child name choices.

After completing the genealogical links, and the status information, we will have potentially the following information for each person in the database

Date of birth, longevity, wealth at death, educational attainment, occupation, birth location, fertility, child mortality, death location, birth order, number of siblings, age at marriage.
